# An organ culture system to model early degenerative changes of the intervertebral disc II: profiling global gene expression changes

**DOI:** 10.1186/ar4301

**Published:** 2013-09-16

**Authors:** Dessislava Z Markova, Christopher K Kepler, Sankar Addya, Hallie B Murray, Alexander R Vaccaro, Irving M Shapiro, D Greg Anderson, Todd J Albert, Makarand V Risbud

**Affiliations:** 1 Department of Orthopaedic Surgery, Thomas Jefferson University, 1015 Walnut Street, Philadelphia, PA 19107, USA; 2 Department of Cancer Biology, Kimmel Cancer Center, Thomas Jefferson University, 233 South 10th Street, Philadelphia, PA 19107, USA; 3 Department of Orthopaedic Surgery, Rothman Institute, 925 Chestnut Street, Philadelphia, PA 19107, USA; 4 Biomet Spine, Biomet Inc., 100 Interpace Parkway, Parsippany, NJ 07054, USA

## Abstract

**Introduction:**

Despite many advances in our understanding of the molecular basis of disc degeneration, there remains a paucity of preclinical models which can be used to study the biochemical and molecular events that drive disc degeneration, and the effects of potential therapeutic interventions. The goal of this study is to characterize global gene expression changes in a disc organ culture system that mimics early nontraumatic disc degeneration.

**Methods:**

To mimic a degenerative insult, rat intervertebral discs were cultured in the presence of TNF-α, IL-1β and serum-limiting conditions. Gene expression analysis was performed using a microarray to identify differential gene expression between experimental and control groups. Differential pattern of gene expression was confirmed using quantitative reverse transcriptase polymerase chain reaction (qRT-PCR) or Western blot.

**Results:**

Treatment resulted in significant changes in expression of more than 1,000 genes affecting many aspects of cell function including cellular movement, the cell cycle, cellular development, and cell death and proliferation. Many of the most highly upregulated and downregulated genes have known functions in disc degeneration and extracellular matrix hemostasis. Construction of gene networks based on known cellular pathways and expression data from our analysis demonstrated that the network associated with cell death, cell cycle regulation and DNA replication and repair was most heavily affected in this model of disc degeneration.

**Conclusions:**

This rat organ culture model uses cytokine exposure to induce wide gene expression changes with the most affected genes having known reported functions in disc degeneration. We propose that this model is a valuable tool to study the etiology of disc degeneration and evaluate potential therapeutic treatments.

## Introduction

Amongst the many potential pain generators in the lumbar spine, symptomatic disc degeneration is thought to be a significant contributor to low back pain (LBP) [1,2] and accounts for more than 25% of lumbar fusion surgery performed in the USA [3]. By some estimates, LBP results in direct and indirect health-care expenditures exceeding $100 billion per year, a considerable portion of which is due to discogenic pain [4]. Despite the scale of this clinical problem, many aspects of the pathogenesis associated with LBP remain incompletely characterized. The highly variable nature of associated symptoms and the presence of multiple potential sources of pain within the spine often confound efforts to accurately identify and study disc degeneration in live subjects. However, most authorities agree that there is a strong link between intervertebral disc degeneration and the LBP [5,6].

Establishing clinically relevant models of disc degeneration has also proven difficult. In contrast to humans, disc degeneration is not commonly seen in most quadrupedal animals, likely due to a variety of factors such as mineral composition, vertebral range of motion, extracellular matrix (ECM) composition, cell type, and weight distribution [7]. Different strategies have been developed to induce disc degeneration in animals including gene silencing, application of supraphysiologic loading, and disc injury. One theoretical advantage of using *in vitro *models to study disc degeneration is the ability to carefully control confounding environmental variables, which may differ between individual animals such as nutrition and the loading environment. To date, most widely used *in vitro *studies are performed with cells isolated from the intervertebral disc, which are then either grown in monolayer or suspended in a matrix that allows cells to assume a three-dimensional phenotype. To capture the benefits associated with *in vitro *models of disc degeneration while maintaining essentially normal cell density and distribution and the native ECM, we have developed and performed initial characterization of a disc organ culture model using intact rat intervertebral discs [8]. Since the hallmark catabolic processes such as matrix breakdown and decreased cellular biosynthesis during human degeneration are mediated by a number of cytokines, including IL-1β and TNF-α [9-14], we chose to mimic the degenerative state by treating organ-cultured discs with a cocktail containing both these cytokines.

Our initial investigations focused on targeted evaluation of a few key molecules known to be intermediaries in the degenerative cascade or essential components of the disc ECM [8]. In the present study, we hope to expand on our initial studies to demonstrate that this model replicates the degenerative phenotype in humans through analysis of global gene expression changes. Our results clearly indicate that this model is a good alternative to *in vivo *and cell-based *in vitro *models of intervertebral disc degeneration.

## Materials and methods

### Disc harvesting and organ culture

The rat tissue samples were obtained under a protocol approved by the Institutional Animal Care and Use Committee (IACUC) of the Thomas Jefferson University (Protocol number 703H). Rat lumbar disc specimens were isolated using a method reported earlier by Ponnappan *et al*. [8]. Briefly, using a sterile surgical blade (number 15) the whole lumbar intervertebral discs (IVDs with the endplates were dissected (*n *= 6 discs/animal) and maintained in organ culture in 12-well cell culture plates. All experimental lumbar discs were cultured in DMEM containing 1% FBS, 10 ng/ml IL-1β (R&D Systems, Inc., Minneapolis, MN, USA), 100 ng/ml TNF-α, 50 μg/ml L-ascorbate, 40 mM NaCl, antibiotics and antimycotics (Cellgro, Mediatech, Inc., Herndon, VA, USA). Control discs were cultured in DMEM containing 10% FBS, 50 μg/ml L-ascorbate, 40 mM NaCl and antibiotics without any cytokines. The discs were maintained in culture for 10 days. The complete medium was replaced every two days for both groups. In each experiment, six lumbar discs from one animal were used per group. The number of animals used for each study is indicated where appropriate.

### Microarray analysis

Total RNA was extracted using the RNeasy Micro kit (Qiagen, Valencia, CA, USA, ) according to the manufacturer's instructions. DNase-treated RNA was quantified on a NanoDrop ND-1000 spectrophotometer (NanoDrop Technologies, Inc., Wilmington, DE, USA), followed by RNA quality assessment by analysis on an Agilent 2100 bioanalyzer (Agilent, Palo Alto, CA, USA). RNA amplification and labeling was performed from 50 ng total RNA by the WT-Ovation Pico RNA amplification system (NuGen Technologies, Inc., San Carlos, CA, USA) as described previously [15]. Each Affymetrix Rat Gene array (RaGene 1.0 ST v1) (Affymetrix, Santa Clara, CA, USA) was hybridized with fragmented and biotin-labeled target for 18 h. Arrays were then washed and stained using Gene-chip Fluidic Station 450, and hybridization signals were amplified using antibody amplification with goat IgG (Sigma-Aldrich, St Louis, MO, USA) and anti-streptavidin biotinylated antibody (Vector Laboratories, Burlingame, CA, USA). Chips were scanned on an Affymetrix Gene Chip Scanner 3000, using Command Console software. Background correction and normalization were done using Robust Multichip Average (RMA) with Gene-spring V 11.5 software (Agilent). A volcano plot was used to identify differentially expressed genes using the unpaired two-sample Student's *t *test, assuming significance for *P *values ≤ 0.05, and fold changes ≥1.5. A heat map was generated by Gene-spring software V 11.5. The differentially expressed gene list was loaded into Ingenuity Pathway Analysis (IPA-8.8) software to perform biological network and functional analyses following a previously described method [16]. In short, IPA was used to convert a list of differentially expressed genes into a set of relevant networks based on a continually updated database of known molecular pathways. This analysis is intended to suggest which cellular processes are most likely to be affected by the gene expression changes taking place in a given experiment. This process generates a network score that reflects the probability that a similar group of genes equal to or greater than the number in the network could be achieved through chance alone. Previous similar analyses have used a score of more than 10 as a cutoff for identifying relevant gene networks [16] with higher values corresponding to networks more likely to be affected by the expression changes seen in the experiment. Four animals for the control and four animals for the experimental group (24 discs/group) were used for this study, *n *= 4 independent experiments. The microarray data can be accessed at the Gene Expression Omnibus number GSE42611 [17].

### RNA extraction and quantitative real-time PCR

The discs were collected and the annulus fibrosus (AF) was separated from the nucleus pulposus (NP) as in previously described method [8]. Total RNA was extracted from NP tissue (RNeasy Micro kit, Qiagen) according to the manufacturer's instructions. The RNA samples were treated with DNase I digestion prior to conversion into cDNA. Single-stranded cDNA was synthesized from 0.5 μg of total RNA using RNA to cDNA EcoDry Premix (Oligo dT) (Clontech Laboratories, Inc., Mountain View, CA, USA) and quantitative (q) RT-PCR was performed using SYBR Green PCR Master Mix (Applied Biosystems, Foster City, CA, USA) on an ABI 7900 HT sequence detection system. A melting curve analysis was performed to ensure primer specificity. The amount of PCR product was estimated using a relative standard curve quantification method. Expression was normalized by using housekeeping gene hypoxanthine phosphoribosyltransferase 1 (*Hprt1*). All the primers used were synthesized by Integrated DNA Technologies, Inc. (Coralville, IA, USA). Sequences for primers are presented in Table 1.

**Table 1:** Primers used for real-time RT-PCR.

Target genes	Sequence ( 5' to 3')	Size (bp)
***CCL20***	**F: **ACCTGATTTGTGTCCCAGTGGTCT **R: **AGCGCCCTTCATAGATTGTGGGAA	175
***IL6***	**F: **AACTCCATCTGCCCTTCAGGAACA **R: **AAGGCAGTGGCTGTCAACAACATC	101
***ADAMTS5***	**F: **GTCCAAATGCACTTCAGCCACGAT **R: **AATGTCAAGTTGCACTGCTGGGTG	147
***MMP-3 ***	**F: **ATTGGCACAAAGGTGGATGCTGTC **R: **ACGACGCCTTCCATGGATCTTCTT	160
***SDC4***	**F: **ACTGAGGTCTTGGCAGCTCTGATT **R: **TACACCAGCAGCAGGATCAGGAAA	80
***Krt19***	**F: **TGGGTGGCAATGAGAAGATCACCA **R: **ATCTTCACCTCCAGCTCGCCATTA	109
***Krt8***	**F: **AGCATCATTGCTGAAGTTCGTGCC **R: **ATCTGGTACATGGTTTCGGCCTCA	80
***Hprt1***	**F: **AGTCCCAGCGTCGTGATTAGTGAT **R: **GAGCAAGTCTTTCAGTCCTGTCCA	139

F, forward primer; R, reverse primer.

### Western blot

Expression of Syndecan 4 (SDC-4) and Keratin 19 (KRT19) were evaluated by Western blot analysis. NP tissue was washed with cold PBS and was extracted with lysis buffer (Cell Signaling Technologies, Beverly, MA, USA) at 4°C for 24 h. The lysis buffer contains: 20 mM Tris-HCl (pH 7.5), 150 mM NaCl, 1 mM Na_2 _EDTA, 1 mM EGTA, 1% Triton, 2.5 mM sodium pyrophosphate, 1 mM β-glycerophosphate, 1 mM Na_3_VO_4_, 1 μg/ml leupeptin, 1 mM PMSF and 1× complete protease inhibitors (Roche, Indianapolis, IN, USA). The tissue lysate was centrifuged for 10 min at 14,000 × g to collect the clear tissue extract. Protein concentration in the extract was determined using a Pierce BCA protein assay kit (Thermo Fisher Scientific Inc., Rockford, IL, USA). Lysates were treated with 0.1 U/ml chondroitinase ABC (Sigma-Aldrich) in 50 mM Tris-acetate EDTA buffer at 37°C for 1 h. Protein extracts (10 μg) were centrifuged at 4°C for 2 min at 14,000 × g and resolved on NuPAGE 4 to 12% Bis-Tris Gels (Invitrogen, Carlsbad, CA, USA). Proteins were transferred onto Immobilon-P Membrane (Merck Millipore, Darmstadt, Germany). The membrane was blocked with 5% nonfat dry milk in PBS with 0.1% Tween-20 and incubated overnight at 4°C in 3% nonfat dry milk in PBS with 0.1% Tween-20 with the appropriate antibody anti-Syndecan 4 (Abcam, Cambridge, UK) and anti-Keratin 19 (Cell Signaling Technologies), respectively. The binding of the secondary antibody was detected by enhanced chemiluminescence (ECL-Plus, GE Healthcare, Little Chalfont, UK). Results were normalized to the content of tubulin detected with monoclonal antibody beta-tubulin (1:3000, Developmental Studies Hybridoma Bank (DSHB)), *n *= 3 independent experiments.

### Senescence-associated β-galactosidase staining

The primary rat NP cells were isolated using a method reported earlier by Wang *et al*. [10]. Cells were plated in Lab-Tek II Chamber Slide System (Nalge Nunc International, Rochester, NY, USA) and treated with 10 ng/ml IL-1β and 10 ng/ml TNF-α for 10 days. The slides were rinsed with PBS, fixed with fixative solution for 15 min at room temperature, and incubated overnight at 37°C with fresh SA-β-gal staining solution (Senescence Detection Kit, Abcam). Cells were observed under a light microscope for development of blue color. The images were captured using a Nikon Eclipse E600 (Nikon, Tokyo, Japan).

### Data analysis

Statistical analysis was performed using Sigma Plot 11.2 statistical software (Systat Software, Inc., Chicago, IL, USA). Differences between groups in continuous variables were analyzed by the Student's *t *test assuming significance at *P *< 0.05.

## Results

### Microarray experiment

**Figure 1:**
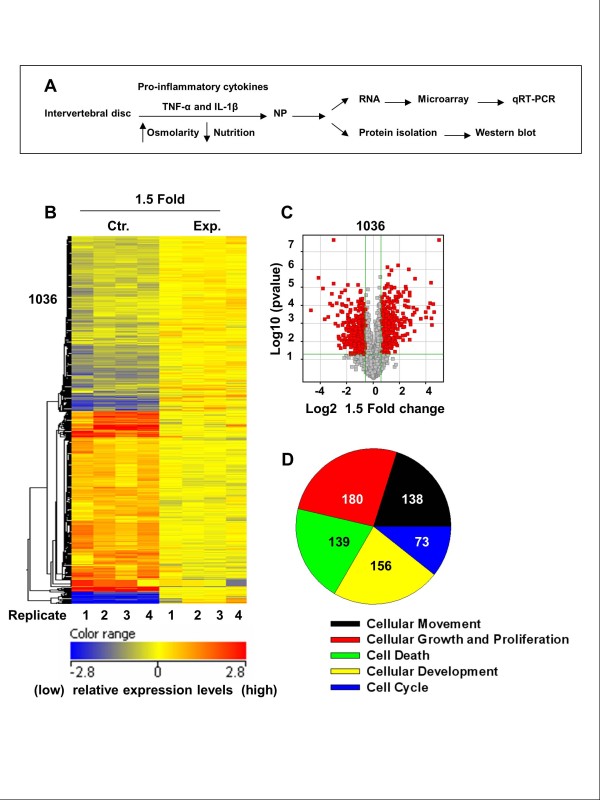
**(A) Schematic showing disc organ culture setup**. Culturing discs under low pO_2_, in hyperosmolar, nutritionally limiting media with exposure to proinflammatory cytokines mimics the molecular changes characteristic of disc degeneration. **(B) **Microarray analysis of rat discs treated with TNF-α and IL-1β for 10 days. The heat map displays gene expression patterns of 1,036 genes after treatment. The relative levels of gene expression are depicted with a color scale, where blue represents the lowest and red represents the highest level of expression. **(C) **Volcano plot of microarray data depicting the significant differences (*P *< 0.05) in expression patterns between the control (Ctr.) and experimental (Exp.) groups, where the red plots represent a 1.5-fold or more difference in expression patterns between the two groups. (**D) **Pie chart showing differentially expressed genes based on major molecular and cellular functions. The total numbers of genes associated with each specific function are listed. Note that some genes may have multiple functions and can be classified in several categories (see Table 2), *N *= 4 independent experiments.

Following organ culture experiment (Figure 1A), RNA was extracted and microarray analysis was performed. Figure 1B depicts the heat map of the significant NP gene expression profile changes between the control (Ctr) and experimental (Exp) groups, demonstrating more than 1,000 individual genes that were either upregulated or downregulated by more than 1.5-fold in the experimental model. In total, 1,036 genes demonstrated a significant upregulation or downregulation based on the microarray results. Figure 1C more clearly depicts the relative balance between genes either upregulated or downregulated by more than 1.5-fold (*P *< 0.05) in the experimental model compared to the control group using a volcano plot; significantly upregulated genes are depicted by red dots to the right of the center while significantly downregulated genes are denoted by red dots to the left of the center. From a functional standpoint, genes changing expression patterns in the experimental model could be divided into several different known cellular functions as seen in Figure 1D: 138 genes affect cellular movement, 73 genes affect the cell cycle, 156 genes affect cellular development, 139 genes are involved in cellular death and 180 genes are known intermediaries in cellular growth and proliferation. Genes contributing to these major cellular functions are shown in Table 2.

**Table 2:** Differentially expressed genes based on major molecular and cellular functions.

*No*.	*Molecular functions *	*No. of molecules*	*P value*
1	Cellular movement	138	1.27E-24 -1.02E-03
***Gene symbol: ***NPM1,FURIN,ZC3H12A,TGFBR1,GAS6,TGFBR3,CCL20,IL6,CCND1,ODC1,VEGFA,CHST2, CTSS,RARA,LUM,FIGF,SERPINE1,GNG12,FGR,TP53,TP53INP1,LCP1,SLC2A1,ERAP1,ITGA5,MMP2,NFKB2, MET,CSF2RB,MIB1,CTSB,ITGA1,CYR61,HMGB2,FLNB,FYN,ADAM17,ICAM1,BMPR2,JAK2,Lyz1/Lyz2,PTPN12, GADD45A,PTPRJ,IGF1,ZFAND5,FABP4,TNFRSF1B,FGF7,GJB2,PLAT,TNFSF11,RHOC,TYROBP,Cd24a,VDAC3, EFNA1,PTGES,GPI,HP,NFIA,ZEB2,CD44,FCER1G,RASGRF1,APBB2,MYH9,MAP3K8,NEDD9,LGALS3,RARRES2, FGL2,S100A4,SDC4,CXCL3,STMN1,CYBB,NOD1,S100A10,GJA1,C3,LCN2,CD93,AQP1,LMNA,PDE4B,GSN, Abcb1b,PPIC,TLR2,CBL,IL1RN,ACTN4,JAG1,MAP2,TMEM176B,SIRPA,IL6ST,RELA,NFIX,MMP14,DDX3X, HIF1A,NFKB1,FAS,PTPRF,ANGPTL4,NFKBIA,GLI3,DDR2,TIMP1,PTPN1,EGLN3,ENPP2,CCRL1,ERRFI1,CHUK, NOS2,ARID5B,CALR,TIMP3,SLC9A3R1,EDNRB,ASAP1,BGN,MAP3K1,BAX,NFKBIZ,FGF1,COL1A1,CXCL16, EBF1,LPAR1,NT5E,CXCL12,CDKN1B,CXCL2,LGALS1			
2	Cellular growth and proliferation	180	1.05E-17 - 5.73E-04
***Gene symbol: ***ST6GAL1,NPM1,FURIN,CRYAB,CTCF,TGFBR1,TCN2,CEP120,GAS6,TGFBR3,MKI67,IL6, CCND1,ODC1,VEGFA,LIFR,SOD2,MT2A,CTSS,RARA,FIGF,OSMR,SERPINE1,HIPK2,TP53,CYP7B1,TP53INP1, NDRG1,ERAP1,MMP2,IER3,NFKB2,MET,CSF2RB,Krt19,ITGA1,CFLAR,MT1E,ADM,FYN,ICAM1,GRB14,DDIT3, GPX1,BMPR2,PSEN2,JAK2,Gp49a/Lilrb4,ID1,ARRDC3,GADD45A,IGF1,PTPRJ,PRRX1,MXD1,CREB1,ASPH, TNFRSF1B,FGF7,IRF2,PPP3CA,PLAT,SERTAD2,TNFSF11,ANGPT1,TYROBP,EGR1,Cd24a,SERPINF1,SMYD2, PTGES,GPI,NOV,KRT8,SRGN,TF,NFIA,SPRY2,PELI1,PTH1R,ZEB2,CD44,FCER1G,RASGRF1,APBB2,MAFB, TCF7L2,LGALS3,RARRES2,MMP3,NDRG2,FGL2,ZFP36L1,MEIS1,GIP,BCL6,CXCL3,TOP1,STMN1,IMPDH2, ITGA11,NUPR1,CYBB,STK17B,TNFRSF11B,SOX4,GJA1,H19,C3,ATF3,LCN2,Abcb1b,MYOF,INHBA,ELF1,TLR2, CBL,IL23R,CXCR7,IL1RN,LIMA1,TGFB3,JAG1,EREG,SIRPA,RBP4,IL6ST,TPM1 (includes EG:22003),ENPEP, RELA,ADAMTS7,ID2,S100A6,MMP14,CLU,SAT1,RPS6KA3,DDX3X,PLA2G2A,HIF1A,NFKB1,TGIF1,FAS, PTPRF,Igh2,HMOX1,AEBP1,SOX9,NFKBIA,ANGPTL4,GLI3,DDR2,PTPN1,TGFB2,ERRFI1,CHUK,NOS2,TERF1, PTGER4,CALR,ARID5B,IL13RA1,MAP3K1,ERO1L,BAX,GRB10,CADM1,FGF1,CTF1,COL1A1,CXCL16,LPAR1, NT5E,CXCL12,SPARC,TSC22D1,SKIL,CDKN1B,CXCL2,LGALS1			
3	Cell death	139	1.09E-12 - 9.48E-04
***Gene symbol: ***NPM1,ST6GAL1,CRYAB,TGFBR1,CTCF,GAS6,SGK1,XDH,SERPINA3,IL6,CCND1,ODC1,VEGFA,BNIP3, SOD2,CTSS,MT2A,DLG4,SERPINE1,HIPK2,FGR,TP53,TP53INP1,SLC2A1,ITGA5,MMP2,NFKB2,IER3,MET, CSF2RB,MIB1,ST3GAL1,CTSB,ITGA1,CFLAR,MT1E,FYN,DDIT3,GPX1,PSEN2,CASP4,JAK2,TANK,ID1,IGF1, GADD45A,CREB1,MXD1,TOP2A,TNFRSF1B,PPP3CA,IRF2,GJB2,PLAT,TNFSF11,RHOC,TYROBP,EGR1,Cd24a, VIM,PLK1,IRAK3,PTGES,GPI,KRT8,SRGN,ENO1,PTH1R,CD44,FCER1G,MAFB,BIRC2,LGALS3,FGL2,GIP, MEIS1,SH3GL2,BCL6,HSPA5,CXCL3,TOP1,STMN1,TNIP1,CYBB,NUPR1,DAG1,NSMAF,BIRC3,STK17B, TNFRSF11B,GJA1,ATF3,LCN2,LMNA,SLC1A1,GSN,INHBA,TLR2,CBL,IL1RN,ZNF274,TGFB3,FBN1,VDAC1, SIRPA,IL6ST,SCD,RELA,ID2,MMP14,CLU,DDX3X,HIF1A,NFKB1,FAS,HMOX1,TGFB1I1,SOX9,NFKBIA,FXN, PTPN1,TGFB2,CHUK,NOS2,PTGER4,TIMP3,BGN,MAP3K1,BAX,CADM1,CTF1,LPAR1,CXCL12,SPARC, RPS6KA5,SKIL,CDKN1B,LGALS1,CASP8AP2			
4	Cellular development	156	6.5E-10 - 9.63E-04
***Gene symbol: ***NPM1,CTCF,TGFBR1,TCN2,RDH10,GAS6,XDH,IL6,CCND1,ODC1,VEGFA,LIFR,SOD2,EIF4G2, RARA,EZR,ZHX2,SERPINE1,PRG4 (includes EG:10216),TP53,NAB1,ERAP1,CREBBP,THY1,ITGA5,IER3,NFKB2, MET,CSF2RB,BSG,MIB1,CTSB,ITGA1,CYR61,HMGB2,ADM,FLNB,FYN,ADAM17,ICAM1,DDIT3,GPX1,BMPR2, PSEN2,JAK2,ID1,IGF1,EFNA5,MXD1,CREB1,SLC11A2,FABP4,HEXB,TNFRSF1B,FGF7,IRF2,TNFSF11,ANGPT1, TYROBP,EGR1,Cd24a,VIM,IRAK3,EFNA1,NOV,KRT8,SERPINH1,SPRY2,PTH1R,CD44,FCER1G,MYH9,MAFB, TCF7L2,MYO1E,RARRES2,LGALS3,MMP3,S100A4,MAP4K4,MEIS1,BCL6,RBP1,BIN1,AGTPBP1,TOP1,ULK2, TNFRSF11B,IGFBP6,GJA1,ATF3,C3,OSTM1,LCN2,LMNA,MYOF,GSN,INHBA,CREB3L2,TLR2,BHLHE41,CBL, IL1RN,TGFB3,JAG1,SIRPA,IL6ST,TPM1 (includes EG:22003),SCD,RELA,ADAMTS7,ID2,RND1,MMP14,DDX3X, EXT1,HIF1A,NFKB1,TGIF1,FAS,HMOX1,TGFB1I1,SOX9,NLK,NFKBIA,ANGPTL4,GLI3,TIMP1,TGFB2,ASPN, EGLN3,ERRFI1,CHUK,NOS2,LONP1,PTGER4,CALR,ARID5B,EPAS1,EDNRB,Acan,ERO1L,BAX,FGF1,CADM1, CTF1,COL1A1,HOPX,EBF1,CXCL12,SPARC,SKIL,COL11A1,CDKN1B,CXCL2,LGALS1			
***No*.**	***Molecular functions ***	***No. of molecules***	***P value***
5	Cell Cycle	73	9.3E-10 - 8.75E-04
***Gene symbol: ***NPM1,CRYAB,TGFBR1,CEP120,GAS6,MEIS1,MKI67,IL6,BCL6,TUBB,CCND1,FBXO4,SOD2, RARA,FIGF,HIPK2,TP53,SETD8,TP53INP1,ATF3,LMNA,INHBA,MET,CCNG2,ZNF274,SIK1,EREG,ADM,RELA, FYN,ID2,TIFA,PKD2 (includes EG:18764),RBBP8,RPS6KA3,DDX3X,HIF1A,JAK2,NFKB1,TGIF1,ID1,SOX9, NFKBIA,IGF1,GADD45A,TIMP1,FSHR,MXD1,TOP2A,E2F5,ERRFI1,TNFRSF1B,NOS2,FGF7,PPP3CA,CALR, TNFSF11,DBI,TYROBP,EGR1,PRC1,PLK1,BAX,GRB10,FGF1,GPI,CXCL12,APBB2,SPARC,MAP3K8,SKIL, CDKN1B,SMARCAD1			

**Figure 2:**
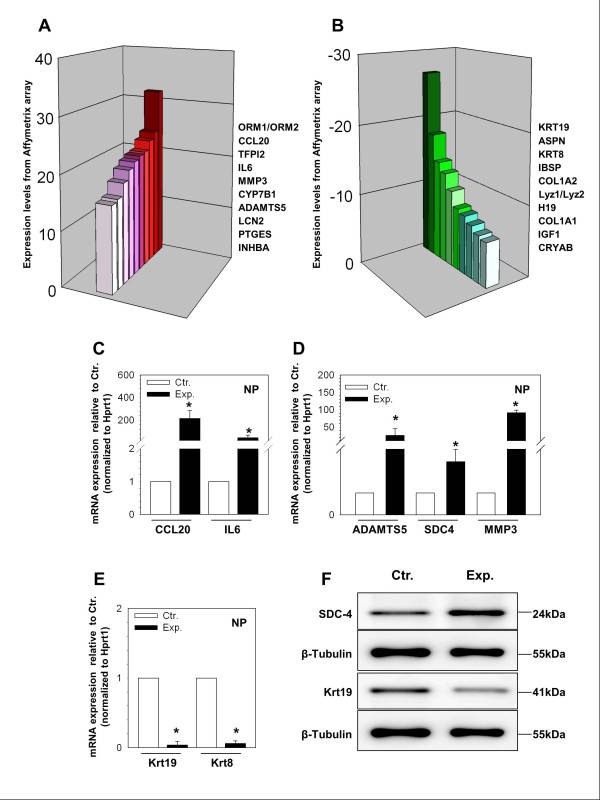
**(A-B) Diagrams illustrating genes with the highest upregulation (A) and downregulation (B) in expression between the experimental (Exp.) and control (Ctr.) discs**. **(C-E) **Quantitative RT-PCR confirms the changes in gene expression identified by microarray analysis between the Ctr. and Exp. discs. Quantitative PCR expression levels were normalized to hypoxanthine phosphoribosyltransferase 1 (*Hprt1*) and shown relative to the Ctr., *N *= 3 independent experiments. **(F) **Western blot analysis of rat nucleus pulposus (NP) tissue lysates shows an increase in expression of Syndecan-4 (SDC4) and a decrease in expression of Keratin 19 (KRT19) in the experimental group, *N *= 3 independent experiments.

The 10 genes most highly upregulated and downregulated in the experimental group are depicted in Figure 2A and 2B, respectively, and the related expression levels are reported in Table 3. Upregulated genes with known roles in disc degeneration included interleukin-6 (*Il-6*), matrix metallopeptidase 3 (*Mmp3*), and a disintegrin and metalloproteinase with thrombospondin motifs 5 (*Adamts5*). Highly downregulated genes with known roles in maintenance of normal disc phenotype or as markers for NP cells included *Krt19*, asporin (*Aspn*), collagen I alpha-1 (*Col1a1*), collagen I alpha-2 (*Col1a2*) and insulin-like growth factor 1 (*Igf1*).

**Table 3:** Top 10 molecules which demonstrated the highest upregulation and downregulation in treated discs.

*Gene symbol*	*Gene description*	*Expression levels*
ORM1/ORM2	Orosomucoid 1	31.714
CCL20	Chemokine (C-C motif) ligand 20	24.260
TFPI2	Tissue factor pathway inhibitor 2	23.074
IL6	Interleukin 6	21.493
MMP3	Matrix metallopeptidase 3	21.267
CYP7B1	Cytochrome P450, family 7, subfamily b,	20.756
ADAMTS5	ADAM metallopeptidase with thrombospondin type 1 motif, 5	19.843
LCN2	Lipocalin 2	18.146
PTGES	Prostaglandin E	15.958
INHBA	Inhibin beta-A	15.593
KRT19	Keratin 19	-26.767
ASPN	Asporin	-17.890
KRT8	Keratin 8	-14.161
IBSP	Integrin-binding sialoprotein	-12.914
COL1A2	Collagen, type I, alpha 2	-10.794
Lyz1/Lyz2	Lysozyme	-8.718
H19	Imprinted maternally expressed transcript	-8.222
COL1A1	Collagen, type I, alpha 1	-7.395
IGF1	Insulin-like growth factor 1	-6.578
CRYAB	Crystallin, alpha B	-6.199

### RNA extraction and quantitative real-time PCR

#### Validation of differentially expressed genes by real-time RT-PCR

Confirmation of microarray data with qRT-PCR was performed for selected genes from the top 10 upregulated and downregulated genes as shown in Table 3. Also included in these confirmatory experiments was *Sdc-4*, as it was also significantly upregulated in the array data and has recently been shown to be a key regulator of *Adamts5 *activity in the IVD [10]. Experimental group demonstrated significant upregulation of chemokine (C-C motif) ligand 20 (*Ccl20*), *Il-6, Adamts5*, and *Mmp3 *by almost 216-fold (*P *< 0.001), 45-fold (*P *= 0.003), 26-fold (*P *= 0.02) and 42-fold (*P *= 0.048), respectively, compared to control (Figure 2C and 2D). Alternatively, *Krt19 *and *Krt8 *were significantly downregulated in the experimental group by 27-fold (*P *< 0.001) and 17-fold (*P *< 0.001), respectively, compared to controls (Figure 2E). *Col1a1 *and *Col1a2 *were not evaluated using qRT-PCR in this study as these genes have previously been shown to be downregulated in experimental discs using the same organ culture model [8]. Notably, the relative changes in gene expression between experimental and control groups were larger when analyzed by qRT-PCR suggesting that microarray analysis may be a conservative evaluation of the gene expression differences between the control and the experimental discs.

### Western blot analysis

Western blot analysis was performed to demonstrate the effect of treatment on the expression of SDC-4 and KRT19. Similar to the results for the microarray and the qRT-PCR experiments, Western blot analysis showed a robust downregulation of KRT19 protein in the experimental group compared to the control group (Figure 2F). There was a strong increase in SDC4 levels in the experimental discs when compared to the control discs (Figure 2F).

### Functional gene networks

**Figure 3:**
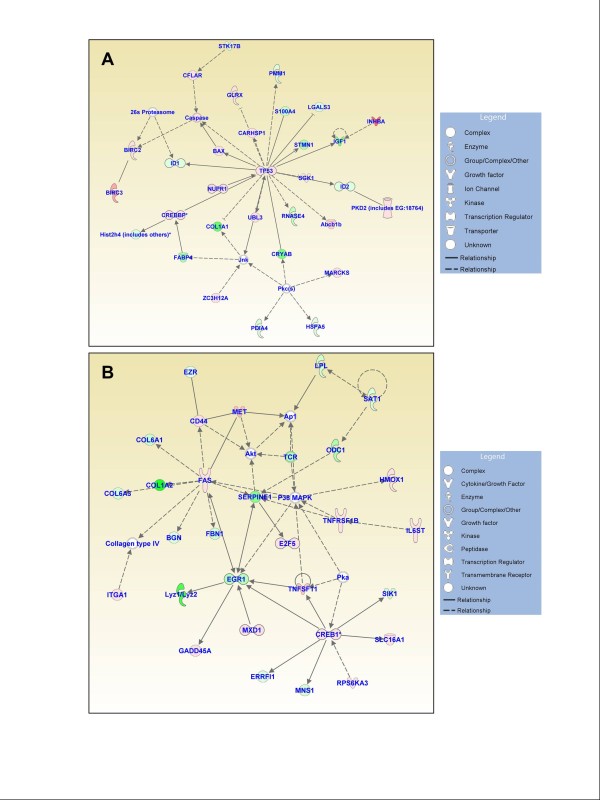
**Functional gene networks identified using Ingenuity Pathway Analysis (IPA) from differentially expressed genes between the control and treated discs**. **(A) **Network-1: cell death, cell cycle, and DNA replication, recombination, and repair (score 31). **(B) **Network-2: cellular growth and proliferation, tissue development, and cancer (score 27). Node color indicates degree of overexpression (red) and the degree of downregulation (green). Colored nodes represent genes of focus, whereas genes in uncolored nodes were not identified as being differentially expressed and were consequently integrated into the networks based on information in the IPA database.

**Figure 4:**
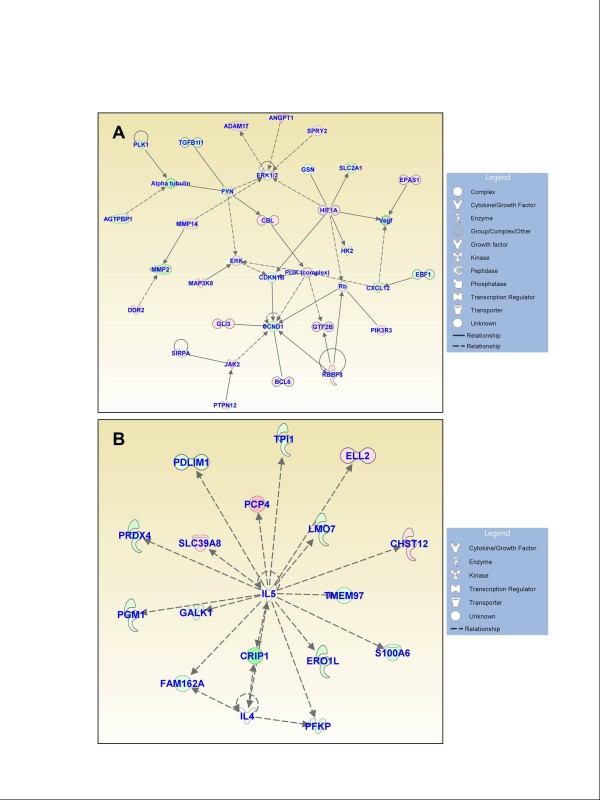
**Functional gene networks identified using Ingenuity Pathway Analysis (IPA) from differentially expressed genes between the control and treated discs**. **(A) **Network-3: cellular development, hematological system development and function, and hematopoiesis (score 27). **(B) **Network-4: carbohydrate metabolism and protein synthesis (score 16).

**Figure 5:**
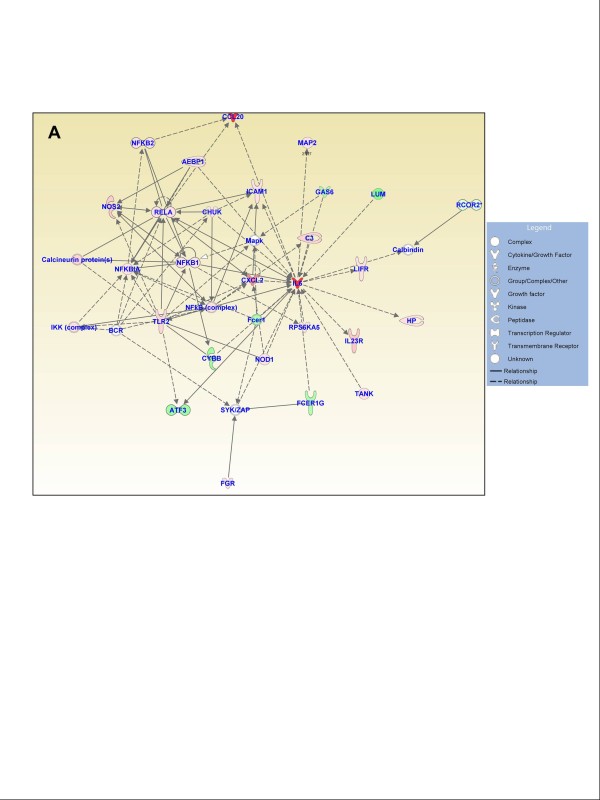
**Functional gene networks identified using Ingenuity Pathway Analysis (IPA) from differentially expressed genes between the control and experimental discs**. **(A) **Network-5: cellular movement, immune cell trafficking, and cell-to-cell signaling and interaction (score 24).

Figures 3, 4, and 5 show the IPA-generated gene networks. The network depicted in Figure 3A is associated with cell death, cell cycle, and DNA replication, recombination and repair, and has a score of 31. The network depicted in Figure 3B is associated with cellular growth and proliferation, tissue development and cancer, and has a score of 27. The network shown in Figure 4A is associated with cellular development and vascular function and development, and has a score of 27. The network illustrated in Figure 4B is associated with carbohydrate metabolism and protein synthesis and has a score of 16. Finally, the network in Figure 5 depicts genes related to cellular movement, immune cell trafficking, cell-to-cell signaling and cellular interactions and has a score of 24.

### Effect of cytokines on NP cell senescence

Staining for SA-β-gal was used to measure the effect of proinflammatory cytokines on phenotype of primary rat NP cells. Increased blue staining was evident in the cytoplasm of the cells treated for 10 days compared to the control group, indicating onset of senescent cell phenotype (Figure S1 in Additional file 1).

## Discussion

Modeling human disc degeneration with a reliable and reproducible animal model is an important step toward further characterization of disc degeneration given the wide inter-individual variation in gene expression, which limits studies on human tissue samples. Furthermore, as our understanding of disc degeneration advances, a reproducible animal model of disc degeneration will enable controlled testing of therapeutic strategies. Many *in vivo *and *ex vivo *models have been proposed to study disc degeneration but most rely on nonanatomic and nonphysiologic methods of inducing degeneration. AF puncture models continue to be a popular method of inducing degenerative changes in quadrupeds to mimic a degenerative phenotype. However, techniques that rely on annular puncture and injection of an enzyme into the disc space to induce degeneration may not accurately model this process in humans [18-22]. Similarly, the use of species that are prone to the development of disc degeneration, such as the sand rat, or other gene knockout models that alter disc biology may not completely reflect the complexities in etiology of human degenerative changes [23-27]. In contrast, an ideal model would not require traumatic injury to the structure of the intervertebral disc and instead induce degeneration using processes native to the IVD. In this investigation, we characterized a rat organ culture model of disc degeneration through analysis of global gene expression using microarray approach that was further validated using quantitative mRNA and protein expression analysis. This nontraumatic model of disc degeneration, based on the use of inflammatory cytokines IL-1β and TNF-α, mimics known gene expression patterns observed in the degenerative human disc. Moreover, this model allows for the evaluation of anabolic, catabolic and inflammatory processes that become imbalanced in disc degradation leading to degradation of the ECM and ultimately to loss of the biomechanical properties of the intervertebral disc.

The phenotypic and genotypic changes associated with disc degeneration are complex, reflecting the interrelatedness of many distinct processes. We found changes in the expression of genes involved in a number of cellular functions; genes controlling cellular growth and proliferation (181 genes) and cellular development (156 genes) were the most frequently affected. Similarly, IPA network analysis demonstrated the highest gene network score amongst genes involved in cell death, cell cycle, and DNA replication, recombination and repair. This finding, and the increased incidence of SA-β-gal in cytokine-treated NP cells, suggests that during degeneration disc cells may be particularly susceptible to processes such as senescence [28], at the same time as identifying genes that are likely to be related to these processes. Furthermore, these IPA networks highlight the web of interrelated pathways that are differentially regulated in discs undergoing degenerative changes; not only may changes in expression of a single gene have wide downstream consequences but these figures hint that the existence of a single key mediator of disc degeneration is unlikely.

Analysis of the top 20 most affected genes indicate that many genes are known mediators of disc degeneration or serve a role in maintaining normal disc function. Prior characterization of gene expression patterns from degenerative human IVD tissue has shown a similar breadth of changes in global expression patterns [29-31]. These gene expression analyses suggest that many different processes are altered in disc degeneration, although most current research tends to focus on the patterns of inflammatory mediator expression. In agreement with recent studies that showed enhanced chemokine-mediated macrophage migration by inflammatory cytokines [32], one of the highly upregulated molecules identified in this study is *Ccl20*. This chemokine is a major regulator of macrophage and mast cells migration and has been reported to be upregulated in other arthritic disorders [33,34]. Gruber *et al*. compared global gene expression profiles between discs with different degrees of degeneration and found broad changes in gene expression patterns, both with respect to the large number of affected genes and the variety of known functions [29]. In contrast to the present study, few of the 47 genes identified by Gruber *et al*. to be differentially expressed had known roles in disc degeneration. In another study evaluating differences between disc tissue harvested immediately post-mortem from patients without known spinal pathology and tissue harvested from patients with degenerative discs, Zhang *et al*. [30] reported more than 500 differentially expressed genes. Consistent with the results from our study, Zhang and colleagues reported a significant upregulation of two known regulators of disc degeneration: *Mmp3 *(3-fold) and *Il-6 *(2.5-fold). Finally, Gruber *et al*. [31] recently utilized microarray analysis and found more than 4,500 differentially expressed genes between early and advanced stages of disc degeneration. Moreover, the authors identified 23 with known roles in the homeostasis of the ECM including collagen subunits, *Aggrecan*, *Adamts *and metalloproteinase inhibitor 3 (*Timp3*).

Certain genes identified in our screen have previously been studied for their role in the pathobiology of intervertebral disc degeneration. Moreover, the similarity with the previously identified expression patterns in human disc degeneration suggests that our model accurately recapitulates natural disc degeneration. IL-6 is an inflammatory cytokine that has been associated with disc herniation and discogenic pain in clinical studies [35,36] but has been less well characterized with respect to its cellular function compared to other inflammatory mediators such as IL-1 or TNF-α. Studer *et al*. [37], recently described contribution of IL-6 in the presence of soluble IL-6 receptor (sIL-6R) in amplification of the inflammatory response to IL-1 and TNF-α in NP cells. The authors suggested that in the NP, IL-6/sIL-R pathway may play a role in potentiating specific functions of IL-1 and TNF-α such as downregulation of matrix production and upregulation of prostaglandins and MMP3. We found that both *Mmp3 *and *Adamts5 *were highly upregulated in our model. MMP3 is an extracellular zinc-dependent proteinase involved in digestion of noncollagen matrix proteins and regulates ECM homeostasis in healthy discs. Upregulation of *Mmp3*, however, is associated with excessive degradation of the ECM, ultimately depleting the hydrophilic nature of the NP and the associated ability to distribute compressive load. Similarly, the ADAMTS peptidases that degrade important proteoglycans including aggrecan [10,38] are shown to be upregulated in degenerative discs by both Le Maitre *et al*. [39] and Pockert *et al*. [40]. SDC4 is a cell surface proteoglycan that has been shown to be essential in activation of the ADAMTS5 within the intervertebral disc, thereby serving as an important regulator of ECM homeostasis [10]. Our study found substantial upregulation of *Sdc4*, confirming the important role *Adamts *play in ECM regulation during disc degeneration. It is important to comment that there is a strong correlation between expression of senescent biomarkers and increased gene expression of catabolic-degrading enzymes in the disc. Thus up-regulation of many of the catabolic enzymes in our model is likely a reflection of senescent cell phenotype as indicated by our SA-β-gal staining [26].

Disc degeneration also affects synthesis of ECM proteins. Collagen I is an important structural protein within the disc, particularly in the AF, which must resist high tensile loads to maintain the disc shape and spine alignment during axial loading. Disc degeneration is typically associated with an upregulation of collagen I that leads to a loss of compliance and hardening of the NP. Interestingly, we found downregulation of two collagen I alpha chains, contrary to what would be expected. Although downregulation of collagen II and upregulation of collagen I is eventually seen in a degenerative IVD, one explanation for these findings is that the initial reparative attempts by the IVD normalize ECM expression patterns, which only later become irrevocably altered.

Downregulation of asporin in the treated cells was also contrary to expectations. Asporin was found to be expressed at higher levels in human discs with more advanced degeneration at both the protein and mRNA level [41]. Furthermore, expression of a particular asporin allele (D14) has been associated with the development of disc degeneration in both Chinese and Japanese populations [42]. Little, however, is known about the molecular basis for these findings as the role of asporin in the IVD has not been thoroughly characterized. Further understanding of the role of this molecule in disc degeneration may provide greater insight into potential explanations for downregulation of asporin in organ culture models.

Other researchers have described organ culture models to study disc degeneration, but used different processes to induce degeneration. Roberts *et al*. [18], Chen *et al*. [19] and Jim *et al*. [43] have all recently described systems that utilize injections of chemolytic enzymes to degrade the ECM and induce a degenerative phenotype. Although these models result in a phenotype consistent with disc degeneration, the homology with the human intervertebral disc degenerative process is questionable as there is little evidence that the nonspecific global chemolysis is the driving force behind human IVD degeneration [29,30,43]. More work is needed to establish whether the use of such enzymes is an appropriate way to induce structural degeneration and inflammation for use in modeling human disc degeneration. Similarly, many organ culture models are based on needle puncture and may be limited by this mechanism of disc injury. Annular needle puncture in animal models and humans results in clinically significant iatrogenic disc degeneration, however, it is unclear whether this model is an accurate representation of disc degeneration commonly seen in humans [44,45] that does not occur via this mechanism. In contrast, induction of disc degeneration using IL-1β and TNF-α mimics the pathways that drive inflammatory processes and disease phenotype in humans.

## Conclusions

Our results clearly demonstrate a widespread differential regulation of genes largely consistent with previous research into the molecular basis of disc degeneration. Furthermore, results from our study found differential expression in many genes known to be mediators of inflammatory processes associated with disc degeneration and regulators of extracellular matrix production. Taken together, these findings suggest that the rat organ culture model described can be used for further studies designed to better understand the etiology of disc degeneration and evaluate potential therapeutic treatments.

## Abbreviations

ADAMTS5: a disintegrin and metalloproteinase with thrombospondin motifs 5; AF: annulus fibrosus; ASPN: asporin; CCL20: chemokine (C-C motif) ligand 20; COL1A1: collagen type I alpha-1; COL1A2: collagen type I alpha-2; Ctr.: control group; DMEM: Dulbecco's modified Eagle medium; ECM: extracellular matrix; EDTA: ethylenediaminetetraacetic acid; Exp.: experimental group; FBS: fetal bovine serum; Hprt1: hypoxanthine phosphoribosyltransferase 1; IL-1β: interleukin-1beta; IL-6: interleukin-6; IGF1: insulin-like growth factor 1; IPA: Ingenuity Pathway Analysis; IVD: intervertebral disc; KRT19: Keratin 19; LBP: low back pain; MMP3: matrix metallopeptidase 3; NP: nucleus pulposus; PBS: phosphate-buffered saline; RT-PCR: reverse transcription-polymerase chain reaction; SDC-4: Syndecan-4; sIL-6R: soluble IL-6 receptor; TIMP3: metalloproteinase inhibitor 3;TNF-α: tumor necrosis factor-alpha.

## Competing interests

TJA receives royalties from Biomet, Inc. (Parsippany, NJ, USA). HBM is employed by Biomet, Inc. (Parsippany, NJ, USA). The other authors declare that they have no competing interests.

## Authors' contributions

DZM and SA carried out the experimental work, analyzed the data and drafted the manuscript.

CKK helped design the studies, secured funding and co-wrote the manuscript. HBM, ARV, IMS, DGA, and TJA helped design the study and prepare the final manuscript. MVR designed the study, helped analyze the data, secured funding and co-wrote the manuscript. All authors read and approved the final manuscript.

## Supplementary Material

Additional file 1 **Figure S1**. Senescence-associated β-galactosidase staining of rat nucleus pulposus (NP) cells following treatment with TNF-α and IL-1β for 10 days. The result indicates that the number of SA-β-gal-positive NP cells is increased in the experimental **(B) **versus the control group **(A)**. Click here for file
