# Supplementary figures and images for: An organ culture system to model early degenerative changes of the intervertebral disc II: profiling global gene expression changes
Source: Arthritis Res Ther. 2013 Sep 16;15(5):R121. doi: 10.1186/ar4301 (PMC3978582; doi:10.1186/ar4301)

## Slide 1
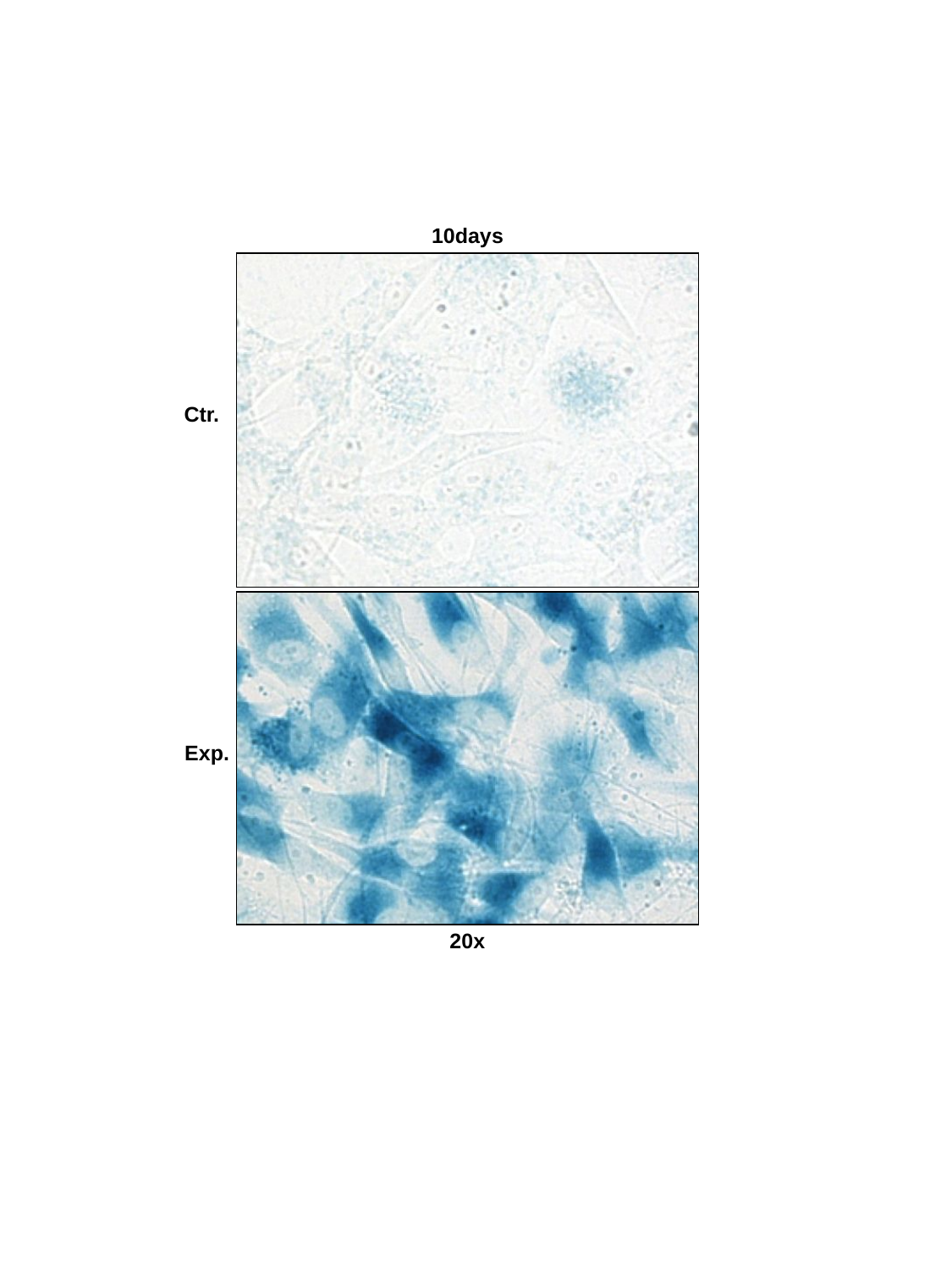

10days
Ctr.
Exp.
20x

Supplement: Additional file 1 — Figure S1. Senescence-associated β-galactosidase staining of rat nucleus pulposus (NP) cells following treatment with TNF-α and IL-1β for 10 days. The result indicates that the number of SA-β-gal-positive NP cells is increased in the experimental (B) versus the control group (A). [file ar4301-S1.PPT]
